# Mitophagy in carcinogenesis, drug resistance and anticancer therapeutics

**DOI:** 10.1186/s12935-021-02065-w

**Published:** 2021-07-05

**Authors:** Yanjie Guan, Yifei Wang, Bo Li, Kai Shen, Quanfu Li, Yingyin Ni, Lei Huang

**Affiliations:** 1 Department of Histoembryology, Genetics and Developmental Biology, Key Laboratory of Cell Differentiation and Apoptosis of Chinese Ministry of Education, Shanghai Key Laboratory of Reproductive Medicine, Shanghai Jiao Tong University School of Medicine, 280 South Chongqing Road, Shanghai, 200025 People’s Republic of China; 2 Innovative Research Team of High-Level Local Universities in Shanghai, Shanghai, People’s Republic of China

**Keywords:** Mitophagy, Mechanism, Carcinogenesis, Drug resistance, Anticancer therapeutics

## Abstract

The mitochondrion is an organelle that plays a vital role in energy production, cytoplasmic protein degradation and cell death. Mitophagy is an autophagic procedure that specifically clears damaged mitochondria and maintains its homeostasis. Emerging evidence indicates that mitophagy is involved in many physiological processes, including cellular homeostasis, cellular differentiation and nerve protection. In this review, we describe the regulatory mechanisms of mitophagy in mammals and yeasts and highlight the recent advances relevant to its function in carcinogenesis and drug resistance. Finally, a section has been dedicated to describing the role of mitophagy in anticancer therapeutics, which is a new frontier that offers a precise and promising strategy.

## Background

The stability of mitochondria is essential for cellular homeostasis and diverse cellular functions [1–6]. Apart from the well-known role of intracellular energy factories through oxidative phosphorylation (OXPHOS) [1, 2], mitochondria are the organelles responsible for the production of reactive oxygen species (ROS), cytoplasmic protein degradation [3], maintenance of calcium homeostasis [4], heme biosynthesis [5], apoptotic activation [6] and innate immunity through mitochondrial antiviral-signaling protein (MAVS) [7].

Autophagy is an important process in cells for degrading proteins and organelles in a lysosome-dependent manner. Mitophagy refers to the process of degrading mitochondria through selective autophagy [8–10]. The concept of mitophagy was first proposed by the Lemasters group in 2005 [8]. Under the conditions of ROS stimulation, nutrition deficiency, and cell senescence, mitochondria undergo membrane potential depolarization [8, 11]. Cellular proteins and depolarized mitochondria are sequestered in autophagosomes. Subsequently, autophagosomes fuse with lysosomes to degrade these contents and maintain the stability of the intracellular environment and mitochondrial fitness [8]. However, under severe conditions, in which mitophagy cannot handle a large number of damaged or dysfunctional mitochondria, cell death pathways are activated, and mitophagy is suppressed [12]. In addition to eliminating damaged mitochondria, researchers have identified numerous physiological and pathophysiological functions of mitophagy. Mitophagy contributes to cell development and erythrocyte differentiation. In mammals, the differentiation of erythrocytes relies on the removal of mitochondria by mitophagy [13]. When knocking out the mitophagy-related gene *BCL2/adenovirus E1B 19 kDa protein-interacting protein 3-like (BNIP3L)* in mice, mitochondria accumulation occurs in erythrocytes, which results in anemia [14]. In adipocytes, mitophagy eliminates excessive mitochondria to promote a beige-to-white adipocyte transition [15]. Since mitophagy maintains mitochondrial homeostasis, it is not surprising that the dysregulation of mitophagy has a role in human diseases. In the brain tissue of Parkinson's disease patients, there are excessive dysfunctional mitochondria, and the mutation of PTEN-induced putative kinase 1 (PINK1) was identified in this tissue [16]. Then PINK1 is proven to be a mitophagy-related gene [17]. Additionally, a similar phenomenon is found in Alzheimer's disease patients, indicating that mitophagy plays a protective role in neurodegenerative diseases [18]. The occurrence and development of cancers is a complicated pathophysiological process, and the effect of mitophagy on cancers will be discussed later.

In this review, we present a brief introduction to the main mechanisms of mitophagy regulation. We also elucidated the roles of mitophagy in carcinogenesis, drug resistance and anticancer therapeutics.

### Common mechanisms of mitophagy

#### Mitochondrial membrane receptors-mediated mitophagy

Mitochondrial membrane receptors mainly include BCL2/adenovirus E1B 19 kDa protein-interacting protein 3 (BNIP3), BNIP3L, FUN14 domain-containing protein 1 (FUNDC1), activating molecule in Beclin 1-regulated autophagy (AMBRA1), FK506-binding protein 8 (FKBP8), ATPase family AAA domain-containing protein 3B (ATAD3B), and some kinds of lipids (cardiolipin (CL) and C18-ceramide). These mitochondrial receptors depend on microtubule-associated protein 1 light chain 3 (LC3)-interacting region (LIR) motifs interacting with LC3 for mitochondrial clearance. They are regulated at the transcriptional or post transcriptional level under hypoxia and starvation conditions by kinases, phosphatases, glucocorticoids and other regulation factors.

BNIP3 and its homologous BNIP3L belong to the BH3-only protein family and induce cell death and mitophagy [19–22]. Upon stress conditions, BNIP3 and BNIP3L are integrated into the outer membrane of mitochondria in the form of a homodimer [23–25]. BNIP3 binds to LC3 by its LIR motif to induce mitophagy in various mammalian cells. Phosphorylation at Ser17 and Ser24 near the LIR motif is important for BNIP3-LC3 interactions [26]. The kinase or phosphatase responsible for the phosphorylation at Ser17 and Ser24 is not yet clear. BNIP3L shares a more than 50% amino acid sequence similarity with BNIP3 [21]. BNIP3L is a mitochondrial receptor mediating mitochondrial elimination during erythrocyte maturation [14, 22, 27]. Under hypoxic conditions or mitochondrial stress, BNIP3L interacts with ATG8 family proteins (GABARAP1/LC3A) through its LIR motif. The interaction between BNIP3L and GABARAP1/LC3A leads to depolarized mitochondrial clearance upon reticulocyte maturation [22, 28, 29]. In BNIP3L-deficient cells, autophagosomal formation is still functional, but mitochondria are unable to fuse with autophagosomes [13, 14, 22, 29] Mitophagy induced by BNIP3 and BNIP3L is regulated by hypoxia-inducible factor 1-alpha (HIF-1α) [30–32]. The upregulation of HIF-1α under hypoxic conditions can enhance the expression of BNIP3 by activating the transcription factor forkhead box O3 (FOXO3) [31]. Glucocorticoids repress PGC1α expression in a glucocorticoid receptor (GR)-dependent manner and mitigate BNIP3L-dependent mitophagy [33].

FUNDC1 plays a distinct role in eukaryotic cells from BNIP3 and BNIP3L. A possible explanation for this might be that FUNDC1 is a mitophagy assistant under hypoxic conditions. FUNDC1 interacts with LC3 through the LIR motif at the cytosol-exposed N-terminus for selective mitophagy to couple with the core autophagic machinery [34]. The phosphorylation of FUNDC1 at Tyr18 in the LIR motif by Src kinase inhibits mitophagy from occurring [34]. FUNDC1 can be ubiquitinated by membrane-associated ring-CH-type finger 5 (MARCH5) and degraded to avoid unnecessary mitochondrial clearance [35]. In addition, kinases such as casein kinase 2 (CK2) and Unc51-like kinase 1 (ULK1) and phosphatases such as phosphoglycerate mutase 5 (PGAM5) regulate the phosphorylation state of FUNDC1 and functionally cooperate to regulate mitophagy [34–37]. Collectively, the phosphorylation state of FUNDC1 dictates its affinity to LC3 and subsequently influences the activation of mitophagy. The PGC-1α-NRF1 pathway is a crucial regulator in mitochondrial biogenesis. PGC-1α and NRF1 also increase the expression of FUNDC1 to enhance mitophagy to promote mitochondrial turnover and maintain functional mitochondria [38]. Such a mechanism serves to maintain the balance between the quality and quantity of mitochondria.

Ambra1 can induce the depolarization of mitochondria which leads to functional mitophagy via a Parkin-independent pathway. Ambra1 binds to the E3 ubiquitin ligase HUWE1 to induce the ubiquitylation of mitofusin 2 (MFN2), a mitochondrial membrane protein, with an overall effect on mitophagy induction. After mitophagy induction, Ambra1 binds to LC3 to complete the autophagosome formation [39]. Ambra1 acts as an alternative mediator in PINK1/Parkin-mutant Parkinson's disease patients [39]. Ambra1 can also be recruited by Parkin during mitochondrial depolarization and activates class III PI3K to form autophagosomes around mitochondria [40].

CL, a kind of lipid located at the inner mitochondrial membrane, is involved in mitochondrial metabolism. Its externalization enables its interaction with LC3 to induce subsequent mitophagy to protect cells from apoptotic cell death. This pathway could be induced by rotenone, staurosporine, and 6-hydroxydopamine [41]. Additionally, Cers1 generates C18-ceramide, a bioactive sphingolipid, and mediates its localization on the membrane of the mitochondria. After mitochondrial fission by dynamin-related protein 1 (DRP1), ceramides interact with LC3B-II to induce mitophagy [42].

**Fig. 1:**
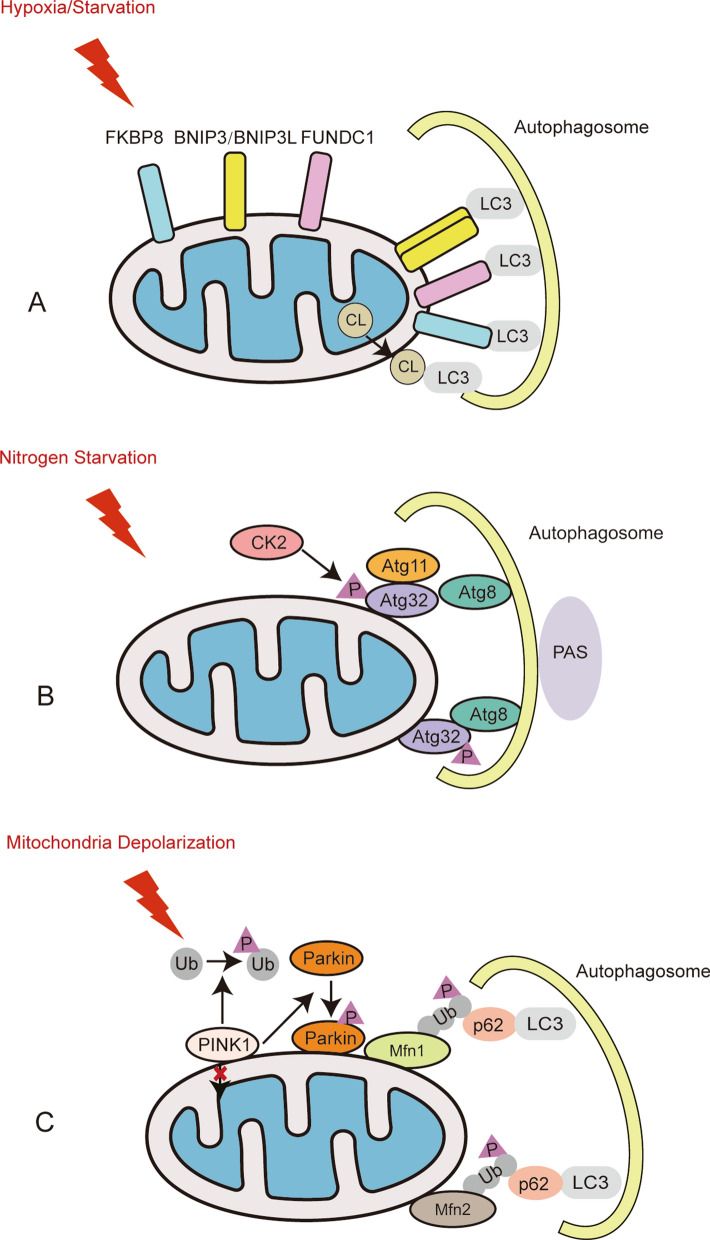
Mechanism of mitophagy regulation in mammals (**A**, **C**) and yeasts (**B**). **A** Mitophagy receptors mediate mitophagy under hypoxia or starvation in mammals. The proteins BNIP3, BNIP3L, FKBP8 and FUNDC1 on the outer member of mitochondria directly bind with LC3 through their LIR domains. Lipid CL externalizes from the inner mitochondrial membrane and interacts with LC3 to initiate mitophagy. **B** Atg proteins mediated mitophagy in yeasts. Under nitrogen starvation, the mitochondrial outer member receptor Atg32 is phosphorylated by CK2 and interacts with Atg8 or Atg11 to promote mitophagy. **C** PINK1/Parkin-mediated mitophagy under mitochondrial depolarization in mammals. Mitochondrial stress blocks the internalization of PINK1. Accumulated PINK1 on the outer member of mitochondria recruits cytosolic Parkin to mitochondria and phosphorylates them. Then, the activated Parkin ubiquitinates the mitochondria outer member proteins such as Mfn1/Mfn2. Adaptors containing LIR motifs (e.g., p62, OPTN, NBR1) recognize these polyubiquitinated proteins and connect to autophagosomes via LC3

FKBP8 is anchored in the outer membrane of mitochondria and acts as a mitophagy receptor. It recruits LC3A by its LIR motif to damaged mitochondria to mediate Parkin-independent mitophagy [43]. ATAD3B, a mitochondrial receptor, interacts with LC3 to induce parkin-independent mitophagy under stress conditions. ATAD3B-induced mitophagy promotes the clearance of damaged mitochondrial DNA (mtDNA) [44] (Fig. 1A).

The phenomenon of mitophagy was first discovered in yeasts in 2005 [8]. Similar to BNIP3/BNIP3L/FUNDC1 in mammals, autophagy-related protein 32 (Atg32) is a mitochondrial receptor protein in the outer membrane of mitochondria with a classic tetrapeptide sequence W/Y/XXI/L/V in yeasts. Under nitrogen starvation, Atg32 is phosphorylated by CK2, especially at Ser114, and then its N-terminus binds to autophagy-related protein 11 (Atg11) [45–48]. The Atg32-Atg11 interaction is crucial for mitochondrial recruitment to the preautophagosomal structure (PAS) [47, 48]. The Atg32 cytoplasmic domain forms a conjugation with autophagy-related protein 8 (Atg8) by its conserved motif to accelerate the process of autophagosomes engulfing the mitochondria. The autophagosomes and lysosomes eventually fuse to clear these contents [49, 50] (Fig. 1B).

#### PINK1/Parkin-mediated mitophagy

PINK1, an outer mitochondrial membrane protein, is sensitive to mitochondrial membrane depolarization. Parkin, named for its causal role in the pathogenesis of early-onset Parkinson's disease, is an E3 ubiquitin-protein ligase comprising five conserved domains, UBL, RING0, RING1, IBR, and RING2 [51, 52]. Under basal conditions, PINK1 is transported from the cytosol to the mitochondrial matrix through mitochondrial translocases, led by its N-terminal mitochondrial targeting sequence. When crossing the inner mitochondrial membrane, PINK1 is cleaved by matrix processing peptidase (MPP), mitochondrial protease presenilin-associated rhomboid-like protein (PARL), and ATPase family gene 3-like protein 2 (AFG3L2). Then the cleaved form of PINK1 is released back to the cytosol and degraded through the ubiquitin–proteasome pathway [53–55]. When mitochondria are compromised and depolarized, PINK1 accumulates on their outer membrane and then recruits Parkin. PINK1 activates Parkin by phosphorylating Parkin on Thr175, Thr217, and Ser65 and thereby initiates mitophagy [56, 57]. Parkin ubiquitinates the mitochondrial proteins of the mitochondrial outer membrane (such as mitofusin 1 (Mfn1) and Mfn2) and promotes ubiquitin chain generation. PINK1 also phosphorylates ubiquitin at Ser65 to enhance the recruitment and activation of Parkin and thus constitutes a feed-forward mechanism to promote mitophagy [58–60] (Fig. 1C).

Several other ubiquitin-protein E3 ligases function in mitophagy in addition to Parkin. An E3 ligase called protein ariadne-1 homolog (ARIH1) is dependent on PINK1 to initiate mitophagy in the absence of Parkin [61]. Seven in absentia homolog 1 (SIAH-1), a conserved ubiquitin E3 ligase, promotes mitophagy by forming the PINK1-synphilin-1-SIAH-1 complex in the absence of Parkin. Synphilin-1 is recruited to the damaged mitochondria by PINK1 to depolarize mitochondria and stabilize uncleaved PINK1. Synphilin-1 then recruits SIAH-1 to ubiquitinate mitochondrial proteins and promotes mitophagy [62].

BNIP3 and BNIP3L also recruit Parkin to the mitochondria. Parkin ubiquitinates multiple mitochondrial membrane proteins, including voltage-dependent anion channel 1 (VDAC1), Mfn1 and mitochondrial Rho GTPase (MIRO), which subsequently interact with p62. The p62 adaptor interacts with LC3 to induce mitophagy [63, 64]. Additionally, PINK1 can be stabilized by BNIP3 in its full-length form, which promotes its ability to recruit Parkin subsequently [65].

Although there are interconnections between these mitophagy pathways, significant differences can be noticed between PINK1/Parkin-mediated mitophagy and mitochondrial receptor-mediated mitophagy. The core mechanism of PINK1/Parkin-mediated mitophagy is the generation of ubiquitin chains recognized by autophagic receptors. However, mitochondrial receptors such as BNIP3 and FUNDC1 contain a conserved LIR motif and directly bind to LC3 on autophagosomes by the LIR motif.

### The role of mitophagy in carcinogenesis, drug resistance and cancer therapeutics

#### Mitophagy and carcinogenesis

Mitophagy plays a multifaceted role in carcinogenesis and cancer progression. Whether it behaves as a tumor promoter or suppressor largely depends on the statuses and subtypes of cancer cells [66, 67]. Mitophagy can remove damaged or dysfunctional mitochondria to maintain the balance between the quality and quantity of mitochondria. After mitophagy, functional mitochondria generate less ROS and limit the tumor-initiating capacity of ROS [68]. On the other hand, once the tumors are already in progress, mitophagy can function as a cytoprotective method to guide tumor progression against chemotherapy-induced apoptosis [69, 70]. Functional mitophagy inhibits the accumulation of damaged mitochondria and prevents carcinogenesis. A study in mouse hepatic cancer reported that thyroid hormone T_3_ restrains carcinogenesis through activating the PINK1/Parkin pathway [71]. The PINK/Parkin pathway can induce mitophagy in liver cells, remove dysfunctional mitochondria and reduce ROS generation. The deletion or mutation of genes such as *PARK2* and *BNIP3* can cause mitophagy inhibition, thereby promoting carcinogenesis and cancer progression [72–75]. Loss-of-function mutations in *PARK2* gene that encodes Parkin, have been detected in human colorectal cancer. The overexpression of Parkin can inhibit the proliferation of colorectal cancer cells. In *PARK2* heterozygous deletion mice, intestinal cancer development is accelerated [72]. FUNDC1-mediated mitophagy also inhibits inflammasome activation and protects against liver carcinogenesis. After the specific depletion of FUNDC1 in liver cells, dysfunctional mitochondria accumulate and therefore trigger inflammasome activation and carcinogenesis [76].

Cancer stem cells (CSCs), which form only a small proportion of the tumor cell population are closely related to the carcinogenesis, invasion, and the drug resistance of cancer. CSCs act as the bottleneck that restricts anticancer therapeutics. Mitophagy serves as a pro-survival pathway for CSCs. For example, in hepatic cancer, mitophagy can maintain the stemness and self-renewal ability of CSCs. Mitophagy can promote p53 degradation, which is combined with mitochondrial clearance. The inhibition of mitophagy leads to p53 transfer into the nucleus and blocks NANOG expression. Without this vital maintaining factor of CSC stemness, the hepatic CSC population is downregulated [77]. ISGylation of Parkin by ubiquitin-like protein ISG15 in pancreatic cancer stem cells (PaCSCs) promotes mitophagy to maintain CSC self-renewal ability. Inhibition of ISG15 could result in reduced Parkin and impaired mitophagy, subsequently impairing PaCSC renewability and tumorigenesis capacity [70]. BNIP3L is highly expressed under hypoxic conditions. BNIP3L-mediated mitophagy promotes glioblastoma survival by clearing ROS and it may play a critical role in CSC maintenance [24].

#### Mitophagy and drug resistance

Certain chemotherapeutic drugs induce mitochondrial dysfunction, produce cytotoxic substances such as ROS, and influence normal metabolic activities [78–81]. Mitophagy is a cytoprotective process in the adaptation to chemotherapy drug treatment. Therefore, targeting mitochondria is regarded as a promising anticancer therapy.

Cisplatin is a widely used platinum-based compound that shows anticancer activity against various cancers. Cancers eventually develop resistance to cisplatin. Therefore, circumventing drug resistance is quite a challenge [61, 69, 82, 83]. Caveolin-1 (Cav-1)/Parkin-mediated mitophagy contributes to the resistance of the non-small cell lung cancer cell line A549 to cisplatin. The cav-1-knockdown A549 cells appear more sensitive to cisplatin because of the downregulated Rho-associated coiled-coil-containing protein kinase 1 (ROCK1) and subsequently suppresses Parkin-mediated mitophagy [82]. Apurinic endonuclease 1 (APE1) plays an important role in the cisplatin resistance of A549 cells, and APE1 is overexpressed in A549 cells and induces Parkin-mediated mitophagy. The knockdown of APE1 restores cisplatin sensitivity and promotes cell apoptosis [83]. In addition, another E3 ubiquitin ligase, ARIH1, is essential for initiating PINK1-dependent mitophagy in the absence of Parkin. ARIH-induced mitophagy acts as a defense mechanism against cisplatin-induced A549 cell death. In ARIH1 knockout cells, cisplatin at the same dose dominantly affects cell growth [61].

A new derivative of betulinic acid (BA), B5G1, has potent anticancer activity toward multidrug-resistant cancer cells by the induction of mitochondrial apoptosis. However, B5G1 can induce mitophagy through the upregulation of PINK1 and subsequent Parkin recruitment. The inhibition of mitophagy by mitochondrial division inhibitor 1 (mdivi-1) or bafilomycin sensitizes drug-resistant cancer cells to B5G1 [81].

Doxorubicin, a DNA damaging agent, greatly influences cell survival by inducing cell death and mitochondrial dysfunction. However, in colorectal cancer, damaged mitochondria are cleared by BNIP3L-mediated mitophagy to reduce oxidative stress and facilitate cell survival. The inhibition of mitophagy by BNIP3L knockout significantly improves its sensitivity to doxorubicin [69].

#### Mitophagy as a target for anticancer therapeutics

Mitophagy promotes cell survival by adapting to stress, but it may lead to cell death due to excessive mitochondrial clearance. Therefore, mitophagy inducers and inhibitors may be equally effective in anticancer treatment.

The inhibition of mitophagy plays a pivotal role in downregulating the drug resistance of cancer cells [78, 84, 85]. In cervical cancer, drug resistance to cisplatin suppresses the efficacy of chemotherapy. Melatonin (N-acetyl-5-methoxytryptamine) is an endogenous indoleamine and a famous antioxidant that reduces hypoxia–ischemia damage and improves sleep. It can also control tumor progression and inhibit mitophagy. Mechanistically, melatonin abates mitophagy by downregulating c-Jun N-terminal kinase (JNK) and subsequently Parkin, and it aggravates cervical cancer cell apoptosis [85]. In hepatic carcinoma, chemotherapeutic drugs such as cisplatin do not perform well in eliminating cancer cells due to inherent mitophagy and autophagy. Cisplatin activates dynamin-related protein 1 (DRP1) to enhance mitophagy. An inhibitor of DRP1-mediated mitophagy (mdivi-1) or a lysosome inhibitor (bafilomycin) increases the susceptibility of hepatic cancer cells to cisplatin rather than directly causing apoptosis [86]. Liensinine, an inhibitor of mitophagy, can markedly increase sensitivity to cisplatin in breast cancer. Liensinine inhibits mitophagy by suppressing the excessive accumulation of autophagosomes, autophagosome-lysosome fusion and the maturation of several important lysosomal hydrolases [87].

Enhanced mitophagy may lead to cell apoptosis due to insufficient functional mitochondria. Enhanced mitophagy can also provide a promising strategy for therapeutic intervention in other cancers. Ketoconazole, an oral antifungal agent, induces PINK1/Parkin-mediated mitophagy by downregulating the expression of COX-2, which promotes the apoptosis of hepatic carcinoma cells [88]. In addition, sorafenib stimulates the apoptosis of liver cancer cells through PINK1/Parkin-mediated mitophagy. It stabilizes PINK1 on the outer membrane of mitochondria and recruits Parkin to dysfunctional mitochondria to initiate mitophagy [89]. Two mitochondria-targeted drugs, Mito-CP and Mito-Metformin, release ULK1 from mTOR-mediated inhibition, decrease mitochondrial membrane potential, and abrogate colorectal cancer cell proliferation through mitophagy [90] (Table 1).

**Table 1:** Mitophagy inhibitors and inducers in anticancer therapeutics

No	Drug	Mitophagy inhibitor or inducer	Mechanisms in anticancer therapeutics
1	Melatonin	Inhibitor	Downregulates c-Jun N-terminal kinase (JNK) and subsequently Parkin, and aggravates cell apoptosis [85]
2	Mdivi-1	Inhibitor	Increases the susceptibility of hepatic cancer cells to cisplatin by increasing mitochondrial membrane permeability [86]
3	Liensinine	Inhibitor	Suppresses the excessive accumulation of autophagosomes, autophagosome-lysosome fusion and the maturation of several important lysosomal hydrolases [87]
4	Ketoconazole	Inducer	Induces PINK1-Parkin mitophagy pathway by downregulating the expression of COX-2 and promotes the apoptosis of hepatic carcinoma cells [88]
5	Sorafenib	Inducer	Stabilizes PINK1 on the outer membrane of mitochondria and recruits Parkin to dysfunctional mitochondria to initiate mitophagy in liver cancer cells [89]
6	Mito-CP and Mito-Metformin	Inducer	Release ULK1 from mTOR-mediated inhibition, decrease mitochondrial membrane potential, and abrogate colorectal cancer cell proliferation [90]

## Conclusions

With deep dissection of its role in carcinogenesis and drug resistance, mitophagy could become a breakthrough in cancer therapy. Whether these mitophagy inhibitors can be employed in clinical therapy for tumors? How to induce apoptosis of tumor cells rather than normal cells by regulation of mitophagy? In addition to endoplasmic reticulum stress, are there other organelles or physiological processes related to mitochondrial autophagy? Are there mitophagy inducers in specific tissues and cell types? How do normal cells sense the precisely regulated dynamic balance of mitochondria? A great deal of literature indicates that maintaining the balance between mitochondrial degradation and accumulation is essential for cellular homeostasis. Therefore, under certain conditions, the inhibition of mitophagy does more harm than good or has a slightly positive effect on the prognosis. As the energy factory of normal cells, the definitive role of mitochondria in cancer merits further systematic investigation. Identifying the precise role of mitophagy will provide an effective approach for mitophagy-based cancer therapy.

To develop new therapeutic strategies for rational anticancer therapy, further studies should pay more attention to the specific mechanisms of mitophagy in CSCs, such as the relationship between the amounts of mitochondria and the maintenance of CSCs. It is important to understand the interactions between oncogenic signaling pathways and mitophagy, the specific role of mitophagy in drug resistance, and the influences of mitophagy on different chemotherapeutic drugs. Mitophagy inducers or inhibitors should be delivered in a more targeted way. At the same time, with the development of novel drug therapies, it is important to be aware of the toxic effects derived from mitochondrial dysfunction and carefully consider them.

## Data Availability

Not applicable.

## Abbreviations

OXPHOS: Oxidative phosphorylation
ROS: Reactive oxygen species
MAVS: Mitochondrial antiviral-signaling protein
BNIP3L: BCL2 protein-interacting protein 3-like
PINK1: PTEN induced putative kinase 1
BNIP3: BCL2/adenovirus E1B 19 kDa protein-interacting protein 3
FUNDC1: FUN14 domain-containing protein 1
AMBRA1: Activating Molecule in Beclin 1-Regulated Autophagy
FKBP8: FK506-binding protein 8
ATAD3B: ATPase family AAA domain-containing protein 3B
CL: Cardiolipin
LC3: Microtubule-associated protein 1 light chain 3
LIR: LC3-interacting region
HIF-1α: Hypoxia-inducible factor 1-alpha
FOXO3: Forkhead box O3
GR: Glucocorticoid receptor
MARCH5: Membrane-associated ring-CH-type finger 5
CK2: Casein Kinase 2
ULK1: Unc51-like kinase 1
PGAM5: Phosphoglycerate mutase 5
Mfn2: Mitofusin 2
DRP1: Dynamin-related protein 1
mtDNA: Mitochondrial DNA
Atg32: Autophagy-related protein 32
Atg11: Autophagy-related protein 11
PAS: Pre-autophagosomal structure
Atg8: Autophagy-related protein 8
MPP: Matrix processing peptidase
PARL: Mitochondrial protease presenilin-associated rhomboid-like protein
AFG3L2: ATPase family gene 3-like protein 2
Mfn1: Mitofusin 1
ARIH1: Protein ariadne-1 homolog
SIAH-1: Seven in absentia homolog 1
VDAC1: Voltage-dependent anion channel 1
MIRO: Mitochondrial Rho GTPase
CSCs: Cancer stem cells
PaCSCs: Pancreatic cancer stem cells
Cav-1: Caveolin-1
ROCK1: Rho-associated coiled-coil-containing protein kinase 1
APE1: Apurinic endonuclease 1
BA: Betulinic acid
Mdivi-1: Mitochondrial division inhibitor 1
JNK: C-Jun N-terminal kinase
